# Influence of Suboptimally and Optimally Presented Affective Pictures and Words on Consumption-Related Behavior

**DOI:** 10.3389/fpsyg.2017.02261

**Published:** 2018-01-29

**Authors:** Piotr Winkielman, Yekaterina Gogolushko

**Affiliations:** ^1^Department of Psychology, University of California, San Diego, San Diego, CA, United States; ^2^Faculty of Psychology, SWPS University of Social Sciences and Humanities, Warsaw, Poland

**Keywords:** affect, priming, emotion, consciousness, behavior

## Abstract

Affective stimuli can influence immediate reactions as well as spontaneous behaviors. Much evidence for such influence comes from studies of facial expressions. However, it is unclear whether these effects hold for other affective stimuli, and how the amount of stimulus processing changes the nature of the influence. This paper addresses these issues by comparing the influence on consumption behaviors of emotional pictures and valence-matched words presented at suboptimal and supraliminal durations. In Experiment 1, both suboptimal and supraliminal emotional facial expressions influenced consumption in an affect-congruent, assimilative way. In Experiment 2, pictures of both high- and low-frequency emotional objects congruently influenced consumption. In comparison, words tended to produce incongruent effects. We discuss these findings in light of privileged access theories, which hold that pictures better convey affective meaning than words, and embodiment theories, which hold that pictures better elicit somatosensory and motor responses.

## Introduction

What is the relationship between affect and cognition? Earlier debates saw some strong competing claims about the separation of the presumed “systems,” primacy of one system over the other, and the minimal processing necessary to trigger affective vs. cognitive reactions (Lazarus, 1984; Zajonc, 1984, 2001). Recent years have witnessed a growing consensus that affective and cognitive processes are tightly intertwined, in terms of both their psychological function and neural substrates, and the effort moved to understand the mechanisms of this connection (Clore and Colcombe, 2003; Pessoa and Adolphs, 2011; LeDoux, 2012; Winkielman et al., 2015a; Barrett, 2017).

In empirical contributions, rather than simply contrasting processing with and without awareness to test for “primacy,” the effort moved on to manipulations of stimulus and task variables that may highlight the affective and the cognitive component of processing. Along with manipulations of stimulus visibility, such manipulations help reveal possible differences in “hot” vs. “cold” contributions, specify the role of consciousness, and examine whether these differences actually matter in terms of actual behavior. In this paper we contribute to this effort by exploring the influence of affective pictures and affective words on participants' spontaneous behavior toward a novel stimulus, and their subjective experience. Before we outline the current studies, we offer some background on the topic of affective influence.

### Affective influence

Affective and emotional influence is a topic of a large and growing literature (for reviews, see Winkielman et al., 2007, 2015b; Lerner et al., 2015). Many studies in this literature examine how exposure to an affective stimulus (e.g., valenced picture) changes evaluative responses to a neutral target that immediately follows (Niedenthal, 1990; Murphy and Zajonc, 1993; Winkielman et al., 1997; Payne et al., 2005). These studies have found that an affective stimulus can influence a subsequent response, with direction of the influence depending on several factors. Many studies report affectively congruent, or assimilative effects—with valence of the prime facilitating responses of similar valence. Thus, in the Fazio et al. (1986) experiments, presentation of a positive word (e.g., wedding) shortened classification of another positive word (e.g., puppy). Similarly, in several experiments, subliminally^1^ presented happy faces, as opposed to angry faces, enhanced ratings of neutral Chinese characters (Murphy and Zajonc, 1993; Winkielman et al., 1997). Such congruent effects are especially likely when primes are subliminal, or supraliminal but unobtrusive (e.g., participants perform some irrelevant task on a visible prime). However, researchers have also observed affectively incongruent, or contrast effects, when the prime facilitates responses opposite of its valence. Such incongruent effects can reflect correction processes, as when the prime is blatant (Murphy and Zajonc, 1993) or evaluatively extreme (Glaser and Banaji, 1999) as well as participants' response strategies (Kiefer et al., 2017). Importantly, these transitions from assimilation-to-contrasts can also be due to automatic mechanisms that operate even with subliminal primes (Hermans et al., 1994), including the dynamics of activation and saturation (Irwin et al., 2010). As such, the study of affective influences is informative about the dynamics of less and more conscious or attentive modes of processing.

### Affective influences on spontaneous behavior

The just mentioned studies explored the influence of affective stimuli on responses that are (i) produced immediately following the prime, and (ii) relatively conceptual (e.g., evaluative categorization or judgment). However, over the last decade or so, there has been a growing interest in influences on behaviors that are (i) temporally distant from the priming episode and (ii) spontaneous and unconstrained. Much of this research involves an unobtrusive activation of a “cold” cognitive concept (e.g., by flashing the word “bet”) and later assessment of spontaneous behaviors (e.g., a decision to gamble). There is a recent debate about these phenomena (Cesario, 2014). Some worry about whether they should be called “priming,” are better characterized as “implicit memory” (e.g., source confusion), or are just a form of “unobtrusive suggestions” and “implicit influence.” Others worry about the empirical robustness of some of these “priming” phenomena. Yet, there is evidence that at least some of such effects can be reliably obtained (Payne et al., 2016).

In our earlier work, we have explored whether exposure to affective, rather than cognitive, stimuli can also influence a spontaneous behavior. Winkielman et al. (2005) exposed participants to a longer series (eight exposures) of subliminally presented happy and angry faces under the disguise of a gender classification task. Following the “priming” (affect induction) episode, participants were given a large pitcher with a novel beverage and were asked to simply pour themselves as much beverage as they wanted and to drink as much as they wanted. We chose this task because of a large literature showing that willingness to explore novel items, including foods and drinks, is a sensitive indicator of the organism's affective state (for review, see Winkielman et al., 2007). As expected, after being primed with happy faces participants poured more beverage and drank more of it than participants primed with angry faces. Interestingly, despite eliciting affect-congruent changes in spontaneous behavior, the primes had no effect on participants' conscious mood (Winkielman and Berridge, 2004; for a similar result see also Winkielman et al., 1997; Zemack-Rugar et al., 2007). Subsequent research has found the impact of visually suppressed emotional faces on a variety of reactions, behaviors, judgments, and decisions (e.g., Sweeny et al., 2009; Bornemann et al., 2012; Almeida et al., 2013; for a review see Axelrod et al., 2014).

### Generality and mechanisms

But, how general are these behavioral effects, and what are their mechanisms? In the current studies we extend our previous research by comparing the influence of briefly and optimally presented affective pictures (faces and scenes) with the influence of valenced-matched words. This comparison can help us to not only understand the parameters of affective influences on behavior, but also may inform the debates about the connection between affect and cognition, and the role of consciousness.

#### Accounts of affective influence

One standard way of thinking about affective influences on behavior is the standard associative memory framework (Bower, 1991). Primes activate valence-congruent material in memory which then guides the interpretation and response to the target stimulus according to principles of applicability (Higgins et al., 1977). This “cold” semantic account can explain some cases of affective influence (Forgas, 2002), but there are also reasons to consider “hot” alternatives. Some arguments for this come from neuroscience research highlighting an important role of affective circuitry in guiding perceptual, attentional, memorial, and action-oriented processes (Phelps, 2005; Pessoa, 2013). More pertinent here are arguments from psychology. One piece of evidence is the relatively greater impact of affective than descriptive dimensions of the same priming stimulus. For example, Murphy and Zajonc (1993) reported that under subliminal exposures, judgments of neutral targets were influenced by the prime's affective dimensions (the valence of facial expression) but not by descriptive dimensions (face gender or age). However, it is possible that these findings are limited to faces, especially to faces presented at short durations due to privileged processing of faces in general (Farah et al., 1998) or affective faces in particular (Öhman, 2002). In fact, other studies found comparable priming effects on lexical decisions with affect- and gender-related words presented at minimal durations (Greenwald et al., 1996). Further, the relative ease of obtaining affective, rather than descriptive priming might sometimes be due to the experimenter's selection of stimuli. Specifically, if affective stimuli form more homogenous sets (i.e., stimuli are clearly positive or clearly negative) than descriptive stimuli, the extraction of the affective dimension will be facilitated (Storbeck and Robinson, 2004). On the other hand, the “hot” interpretation of Murphy and Murphy and Zajonc's (1993) effects is consistent with recent evidence that autistic participants (with atypical affective functioning) do not show the typical advantage in processing affective, as opposed to gender, information from faces (Clark et al., 2008). Finally, the “hot” account is supported by observations that affective stimuli can sometimes influence behaviors that tap a person's affective state, but not descriptive measures that tap accessibility of semantic concepts. For example, as mentioned earlier, Winkielman et al. (2005) observed priming effects on hedonic behavior (pouring and drinking), but not on mood ratings. Presumably, if the underlying process involved semantic activation of affective concepts, then exposure to affective primes should have also influenced participants' responses on descriptive measures (Higgins et al., 1977). However, it is possible that some behavioral measures are more sensitive to priming (cognitive or affective) than more descriptive measures, such as self-reports.

#### Embodied accounts

Recently, a different perspective on phenomena of cognitive and affective influence has been offered by the framework of embodied or grounded cognition (Barsalou, 1999, 2010; Niedenthal et al., 2005; Niedenthal, 2007; Winkielman et al., 2015b). This framework acknowledges that processing can proceed in a “disembodied” fashion, relying solely on the semantic memory system. However, processing can also be “embodied,” where perceivers recruit somatosensory states that occur during the actual experience with objects in the world. Importantly, the degree of “embodiment” depends on the nature of the stimulus (e.g., perceptual vs. conceptual) and task demands (e.g., shallow vs. deep processing). That is, stimuli vary in the degree they activate somatosensory resources, and response production can also differentially draw on somatosensory resources (Barsalou, 2010). Translated into the domain of affective influence, the embodiment framework suggests that affective influence can occur in a “disembodied” way (i.e., involve only activation of relevant evaluative concepts) or in an “embodied” way (i.e., involve engagement of genuine affective reactions). One interesting empirical prediction from this account is that affective stimuli that recruit an embodied response should exert greater influence on certain kinds of behavior than comparable affective stimuli that do not recruit an embodied response. Specifically, stimuli that elicit somatosensory reactions should influence behaviors that require participants to engage in an “embodied simulation,” and thus draw on the primed somatosensory resources. In contrast, behaviors that do not require embodied simulation should not be influenced (see Kan et al., 2003; Solomon and Barsalou, 2004). For example, one should see embodiment effects on processes that lead to action to be taken with the stimulus (e.g., deciding how much to pour and drink), but not on purely conceptual responses (e.g., simple ratings).

### Current studies

The current studies sought to compare the influence of affective pictures and words presented at different durations on behavior using the procedure from Winkielman et al. (2005). Specifically, we predicted that pictures would have a stronger influence on consumption behavior than matched emotional words. This prediction was based on two considerations.

The embodiment approach predicts greater influence of pictures because bodily responses are more easily engaged by affective information presented by pictures rather than by written words. For example, Larsen et al. (2003) found stronger electromyographic (EMG) responses to pictures (IAPS set) than words (ANEW set), even though the stimuli were matched in self-reported valence and arousal (Bradley and Lang, 1999; Lang et al., 2005). Similarly neuroimaging studies show that pictures of emotional scenes and emotional facial expressions induce robust activations in the amygdala and related structures (Hariri et al., 2002; Norris et al., 2004). In fact, facial expressions elicit these activations even when presented unobtrusively (Critchley et al., 2000) or unconsciously (Whalen et al., 1998; Morris et al., 1999). In contrast, studies with emotional words reveal much weaker effects, unless researchers use very strongly charged stimuli (Isenberg et al., 1999) or very sensitive measurement techniques, such as intracranial recording directly in the amygdala (Naccache et al., 2005).

Another reason why pictures should have greater influence than words comes from the literature suggesting that pictures have a privileged access to the cognitive system (Potter et al., 1986; Glaser, 1992), including the network representing affective information (De Houwer and Hermans, 1994). Consistent with this assumption, several cognitive studies found that pictures are more effective as primes, and a recent study found a stronger impact of pictures than words in an evaluative priming task (Spruyt et al., 2002). We come back to the issue of similarities and differences between the embodiment and cognitive access account in the discussion.

We examined the differences between pictures and words in two studies. In both studies, the primary manipulation was the valence (positive vs. negative) and format (picture vs. word) of the affective prime. Specifically, Experiment 1 compared the impact of emotional facial expressions vs. valence-matched emotional words, whereas Experiment 2 compared the impact of pictures of various emotional objects to valence-matched words representing the same objects. For both experiments, we predicted greater behavioral impact of affective pictures than words. The experiments also examined several other issues, as follows.

We also wanted to examine the role of awareness and processing amount on the impact of pictures and words. As discussed earlier, on some views awareness may lead to a reversal of affective influence—switching from assimilation to contrast. Alternatively, greater awareness could enhance the impact of the stimulus due to greater processing. Finally, we could observe comparable effects due to early nature of processing of emotional facial expressions (e.g., Critchley et al., 2000).

The processing manipulation also speaks to possible concerns that picture stimuli are easier to physically perceive or comprehend than word stimuli, thus explaining their greater effects on behavior (Glaser, 1992). To address these questions, we added two manipulations. In Experiment 1, we manipulated stimulus duration, with some stimuli presented subliminally and other stimuli supraliminally (with priming made unobtrusive by focusing participants on a valence-irrelevant dimension). In Experiment 2, we manipulated stimulus frequency, with some stimuli representing highly frequent, easily recognizable everyday objects and other stimuli representing less frequent objects. If the difference in behavioral impact of pictures and words holds across duration and frequency manipulations, this suggests that the difference cannot be simply due to perceptibility or comprehension.

Finally, in both experiments, we measured participants' motivational state (hunger and thirst). Our earlier research found that affective priming of consumption behavior with facial expressions was stronger for thirsty participants (Winkielman et al., 2005). Thus, we sought to examine whether Experiment 1 would replicate this effect using facial expressions, and whether Experiment 2 would yield a similar effect using emotional scenes (e.g., dog, gun) which, unlike facial expressions, are less likely to be processed via low-level circuitry and might be less directly tied to approach-avoidance.

The primary dependent measure in both experiments was consumption behavior—the amount of a novel beverage that participants poured and drank. We selected consumption behavior for several reasons. First, earlier work in our lab and other labs showed that consumption behavior is sensitive to manipulation of affective state (Laeng et al., 1993; Strahan et al., 2003; Winkielman et al., 2005). Second, it is important to explore the impact of affective priming on a relatively consequential behavior (a voluntary decision to ingest a novel beverage of uncertain taste and composition. Furthermore, drinking allows one to assess the impact of affective priming with “real-world” units such as volume and price. Finally, as in earlier studies, we also measured changes in subjective experience—mood and arousal, but because our previous studies found no effects on these measures, we predicted no differences here. We return to this issue later.

## Experiment 1

This study compared the impact of subliminal and supraliminal facial expressions and valence-matched words on consumption behavior and measures of subjective experience. We expected that facial expressions would have a stronger impact than words regardless of presentation duration.

### Method

#### Participants and procedure

The study was approved by UCSD Human Research Protection Program. Fifty undergraduates (19 males, 31 females, mean age = 21.1 years) participated for extra credit. Five additional participants were run, but they were replaced because they reported on a post-experimental questionnaire that they saw the subliminal primes (see below). Participants were told that the study involved a computer task, a tasting task, and a rating task, all performed in individual rooms outside the view of the experimenter. The sequence of experimental events, explained in detail shortly, was as follows. First, participants completed a consent form and a pre-task questionnaire. Second, the participants performed four iterations of a classification task on a computer and the beverage tasting and rating task. Finally, participants filled out a post-experimental survey and were thanked and debriefed.

#### Pre-task questionnaire

The pre-task questionnaire assessed participants' baseline affective and motivational state with the following questions: “How hungry are you at the moment?” (0 = not at all, 10 = extremely), “How thirsty are you at the moment?” (0 = not at all, 10 = extremely), “How many hours has passed since your last meal?” (0.5–10 h), “How large was your meal?” (1 = snack, 3 = large), “How much of a sweet tooth do you have?” (0 = not at all, 10 = very much), “How do you feel right now, at this very moment?” (−5 = unpleasant, 5 = pleasant), “How much arousal do you feel right now, at this very moment?” (−5 = low, 5 = high).

#### Classification (priming) task

Participants were exposed to affective primes under the disguise of a categorization task. We told participants that they would see different stimuli quickly presented in succession, and should ignore all but the last stimulus. If the stimulus was a face, participants judged whether it was male or female, and if it was a word, whether it was an animal or an object.

**Figure 1:**
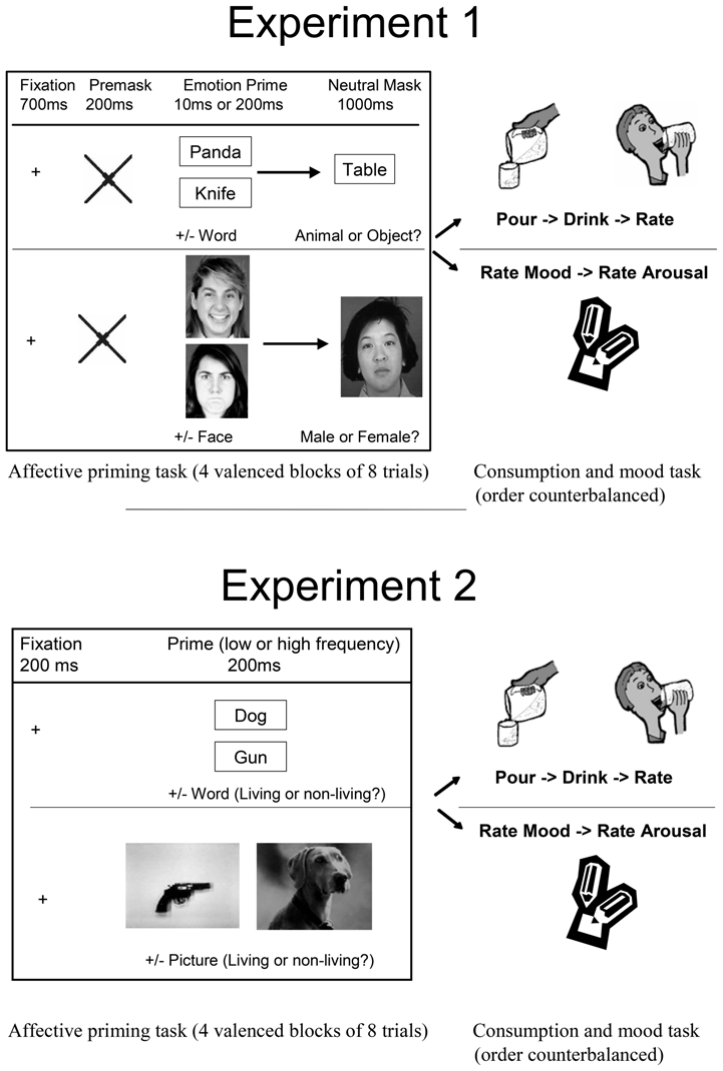
Sequence of events in Experiment 1 (top panel) and Experiment 2 (bottom panel).

The structure of a trial was as follows (Figure 1, top panel). Each trial started with a small 700-ms fixation cross, followed by 200-ms large cross that served as a pre-mask. The prime was then presented for either 10 ms (subliminal) or 200 ms (supraliminal). Finally, the prime was followed by a 1,000-ms neutral post-mask, which participants were to classify as quickly as possible. After one block of eight trials, participants received the consumption and rating task. In total, there were four blocks of valenced primes, counterbalanced across subjects: (i) positive words, (ii) negative words, (iii) positive faces, and (iv) negative faces. The duration of the prime was manipulated between subjects.

#### Primes and mask stimuli

Face stimuli came from the JACFEE series (Matsumoto and Ekman, 1988). Affective primes were 8 negative and 8 positive faces and masks were 16 neutral faces. Half of the faces were Caucasian and half Asian, half male and half female. All faces were shown in black and white and their size was approximately 15 cm^2^. Word stimuli came from the ANEW set (Bradley and Lang, 1999) and from a set developed by Storbeck and Robinson (2004). Affective primes were 8 positive and 8 negative words and masks were 16 neutral words. Half the words were of animals and half were of objects. All word stimuli were in black font against a white background with a size of approximately 8–15 cm wide and 2.5 cm high. Images were shown on a 48-cm monitor, approximately 50 cm away from the participant. The monitor and graphics card were capable of supporting 100 Hz refresh rate which allowed for presentation of 10 ms stimuli (as limited by precision of E-Prime software).

All facial and word stimuli were matched on valence (within 0.5 SD) based on a pre-test in which 16 participants rated a larger set of stimuli on a 1–9 scale (1 = extremely negative, 9 = extremely positive). The exact stimuli and mean ratings are reported in Appendix 1 (left panel).

#### Beverage task

After each round of the classification task, participants were asked to pour and drink the beverage, and to rate their mood and arousal, with task order counterbalanced across subjects. Finally, they were asked to rate the beverage using four questions: “How delicious is the drink?”(0 = not at all to 10 = extremely delicious), “How much of this drink would you like to drink right now?”(0 = none to 6 = pints), “How much would you pay for the drink?”(1 = $0.10 to 10 = $1.00), and “How sweet is this drink?” (0 = not at all to 10 = very much).

As in previous studies, the beverage was made out of Kool-Aid lemon-lime flavored powder, water, and sugar (see Winkielman et al., 2005 for details). To ensure that the beverage was unfamiliar for each condition, we varied sugar and powder proportions. We used four 2-liter pitchers (labeled A, B, C, and D) that contained approximately 600 ml of the beverage at the beginning of the study. Participants poured the drink into 250-ml cups (labeled A, B, C, and D). After each participant left, we used an electronic scale to weigh the amount of beverage left in the pitcher and in the cup, which allowed us to determine how many grams of liquid the participant poured and drank.

#### Feelings rating task

The feelings rating task included the following questions: “How do you feel right now, at this very moment?” (−5 = unpleasant to 5 = pleasant), “How much arousal do you feel right now, at this very moment?”(−5 = low, 5 = high). The feelings rating task also included a supplemental measure that asked participants to rate 22 emotions currently felt (1 = not at all to 5 = extremely) using a PANAS scale supplemented by 2 items (happy and angry). These data are not reported for Experiment 1 because they were unfortunately lost due to experimenter error. However, in previous studies using this supplemental measure, and in Experiment 2, we found no significant effects.

#### Post-experimental survey

The final questionnaire tested participants' impression of the experiment and tested for any suspicions about the experiment priming or consumption tasks. Participants were asked about any unusual aspects of the procedure, any briefly flashed pictures, the drinks, and changes in their mood.

### Results

We analyzed how valence of the prime (positive vs. negative), format of prime stimulus (faces vs. words) and prime duration (subliminal vs. supraliminal) influenced measures of (i) consumption behavior, (ii) subjective experience, and (iii) drink ratings. The relevant means are presented in Table 1. Data underlying these analyses are available under the following link: http://pages.ucsd.edu/~pwinkiel/koolaid-picture-words.zip.

**Table 1:** Means and standard errors of critical dependent measures in Experiment 1.

**VALENCE**	**Positive**				**Negative**			
**FORMAT**	**Faces**		**Words**		**Faces**		**Words**	
**DURATION**	**Supra**	**Sub**	**Supra**	**Sub**	**Supra**	**Sub**	**Supra**	**Sub**
**BEHAVIOR**								
Pouring	56.96	48.62	46.42	43.85	46.17	44.31	55.54	42.85
	*8.58*	*8.24*	*7.09*	*6.81*	*6.50*	*6.24*	*6.43*	*6.17*
Drinking	41.33	33.81	33.71	28.04	31.71	26.88	37.04	34.08
	*7.24*	*6.96*	*5.52*	*5.31*	*5.41*	*5.20*	*6.02*	*5.78*
**EXPERIENCE**								
Mood	1.67	0.88	1.67	1.15	1.79	1.00	1.75	1.04
	*0.38*	*0.36*	*0.37*	*0.36*	*0.38*	*0.37*	*0.31*	*0.30*
Arousal	−0.75	−1.54	−0.63	−1.27	−0.79	−1.58	−0.67	−1.23
	*0.51*	*0.49*	*0.50*	*0.48*	*0.48*	*0.46*	*0.51*	*0.49*
**DRINK RATINGS**								
Delicious	5.29	4.96	5.42	5.00	5.42	5.15	4.71	5.54
	*0.39*	*0.38*	*0.44*	*0.43*	*0.49*	*0.47*	*0.34*	*0.33*
Wanting	1.42	1.38	1.42	1.62	1.29	1.19	1.29	1.50
	*0.22*	*0.21*	*0.21*	*0.20*	*0.20*	*0.19*	*0.20*	*0.19*
Paying	0.40	0.42	0.43	0.43	0.41	0.40	0.38	0.42
	*0.05*	*0.04*	*0.05*	*0.05*	*0.05*	*0.05*	*0.05*	*0.04*
Sweet	5.29	5.73	5.42	5.73	5.71	5.31	5.25	6.08
	*0.38*	*0.36*	*0.44*	*0.42*	*0.45*	*0.44*	*0.42*	*0.41*

*Consumption behavior was measured in grams. Experience on −5 to + 5 scale (not at all, very much). Ratings of deliciousness and sweetness on 0–10 scale. Wanting on 0–6 scale reflecting desired amount, and paying on 10 cents to 1 dollar scale*.

#### Consumption behaviors: pouring and drinking

We tested the influence of priming on two different types of consumption behavior: pouring and drinking. These two DVs were analyzed with a four-way mixed MANOVA with three within-subject factors: behavior type (pouring and drinking), prime valence (positive and negative), prime format (faces and words), and a between-subject factor of prime duration^2^. This MANOVA found no effect of prime duration and a theoretically uninteresting main effect for behavior type, showing that participants poured more than they drank, *F*_(1, 48)_ = 41.30, *p* < 0.001. More interesting, there was a valence by stimulus format interaction, *F*_(1, 48)_ = 4.56, *p* = 0.038. To understand this interaction we ran MANOVAS testing separately the impact of pictures and the impact of words (with all other factors included). The MANOVA on pictures revealed that consumption behaviors (pouring and drinking) were greater after positive than negative faces, *F*_(1, 48)_ = 5.25, *p* = 0.026. In contrast, the MANOVA on words revealed that the impact of valence was not significant (*F* = 1.04), with the direction of means suggesting a decrease of consumption behavior after positive words.

**Figure 2:**
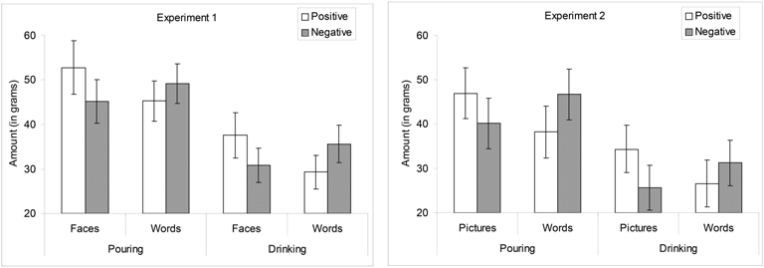
Amount poured and consumed as a function of valence and stimulus type. Experiment 1 (left panel). Experiment 2 (right panel). Error bars are + 1/−1 standard errors.

The above analysis did not reveal a significant three-way interactions involving behavior type (pouring vs. drinking). However, as discussed shortly, these two measures were differently impacted by thirst. Furthermore, we wanted to probe the nature of the priming effect on each individual measure of consumption behavior. We also dropped duration from these additional analyses, as we found no effects earlier (and in later analyses). The results are illustrated in Figure 2 (left panel). On pouring, the 2-way interaction of valence and stimulus format was marginal, *F*_(1, 49)_ = 3.06, *p* = 0.087. Simple effects analysis revealed that participants tended to pour more after positive than negative faces, *t*_(49)_ = 1.7, *p* = 0.095, with no significant difference after positive than negative words (*t* > 1). On drinking, the valence by stimulus format interaction was also marginal, *F*_(1, 49)_ = 3.91, *p* = 0.054. Simple effects analysis revealed that participants drank more after positive than negative faces, [*t*_(49)_ = 2.07, *p* = 0.043], and tended to drink less after priming with positive than negative words, *t*_(49)_ = 1.07, *p* = 0.29.

##### Effect of thirst

Our previous work showed that priming with emotional faces had the strongest effect on thirsty participants (Winkielman et al., 2005). Therefore, we conducted an analysis with 5 factors. Three within factors: behavior type (pouring and drinking), prime valence (positive vs. negative), and stimulus format (picture format). Two between factors: duration (subliminal, supraliminal), and thirst (high vs. low, determined by a median split). This analysis again revealed a main effect of behavior type, *F*_(1, 46)_ = 39.64 (pouring higher than drinking), a 2-way interaction of valence and format, *F*_(1, 46)_ = 3.78, *p* = 0.058, a 3-way interaction of valence, format and thirst, *F*_(1, 46)_ = 6.75, *p* = 0.013, and a 4-way interaction of behavior type, valence, format, and thirst, *F*_(1, 46)_ = 7.285, *p* = 0.01. Duration did not enter in any effects so it was dropped from subsequent analyses designed to further understand the patterns. To make things simpler, and because behavior type interacted with other factors, additional analyses were conducted separately on drinking and on pouring.

On drinking, there was a 3-way interaction of valence, stimulus format, and thirst, *F*_(1, 48)_ = 14, *p* < 0.01. We decomposed this interaction next by separate analyses of valence and thirst on different prime formats. Using only faces, the analysis revealed a 2-way interaction of valence and thirst, *F*_(1, 48)_ = 7.69, *p* < 0.01. Thirsty participants drank more after positive faces than negative faces, [*t*_(27)_ = 3.00, *p* < 0.01]. For non-thirsty participants, facial valence did not significantly influence drinking (*t* < 1). Using only words, the analysis yielded a 2-way interaction of valence and thirst, *F*_(1, 48)_ = 7.60, *p* < 0.01. Interestingly, the simple effects were in opposite direction to the effects of faces. Thirsty participants drank less after positive words than negative words, *t*_(27)_ = −2.37, *p* < 0.05. This contrast effect was unexpected and we will return to it in the discussion. For non-thirsty participants, word primes did not influence drinking, *t* < 1.7.

Because of possible distortions inherent in median-split analyses of continuous variables, we also correlated participants' level of thirst with a change score reflecting the impact of the prime on their drinking behavior (the within-subject difference in drinking after positive vs. negative primes). Consistent with the median-split analyses, greater thirst predicted greater congruent impact of facial primes, *r*_(50)_ = 0.36, *p* < 0.05, and greater incongruent impact of word primes, *r*_(50)_ = −0.30, *p* < 0.05.

Finally, no significant main effects or interactions with thirst were obtained on pouring. Continuous measure of thirst also did not predict the impact of facial or word primes on pouring.

#### Ratings of subjective experience and ratings of drinks

Priming effects on subjective experience and ratings of drinks were analyzed with a three-way ANOVA with the within-subject factors of prime valence, prime format, and duration. On the mood rating, there were no significant effects. On arousal, there was an unexpected main effect for the stimulus type, with participants exposed to words reporting higher arousal ratings, *F*_(1, 48)_ = 7.92, *p* = 0.01. For ratings of drinks, there were no significant effects, as in earlier research involving similar “pouring and drinking” procedure (Winkielman et al., 2005, Study 1).

Finally, as above, we redid the same analyses adding thirst. There were no significant thirst effects on ratings of mood and drinks, and no significant interaction between valence of the prime and thirst (all *F*s < 1).

### Discussion

Experiment 1 obtained results that are reasonably consistent with our predictions and earlier work. Facial expressions influenced consumption behavior in a valence-congruent manner, with participants drinking more after positive (happy) than negative (angry) faces. As in earlier work, the influence of facial expressions was amplified by thirst. As previously, we found no significant effect of facial expressions on subjective experience, though obviously proving the null effect of faces on subjective experience would require evidence for H0 (Bayes factor favoring the absence of an effect) ideally with much larger samples. So, for now the conclusion is very tentative.

Surprisingly, we did not find significant main effects or significant interactions involving stimulus duration—whether the primes were subliminal (near threshold) or supraliminal (visible, though unobtrusive). Though unexpected, this echoes some reports of comparable behavioral effects for subliminal and visible but unobtrusive primes (Bargh, 1992) and some neuroimagining findings of comparable activation effects for subliminal and unobtrusive presentations of emotional facial expressions (Critchley et al., 2000). But again, we would need stronger evidence to confidently claim the absence of the duration effect.

Interestingly, in comparison with faces, words tended to have a valence-incongruent influence. There was one significant contrast effect on drinking for thirsty participants, but other contrast effects were only at the level of a tendency. We will return to this observation in the discussion, though obviously this fragile pattern needs to be replicated.

Finally, on ratings of subjective experience, participants reported greater arousal after words than faces. However, this non-intuitive finding should be interpreted with caution as we only balanced the words and faces on pretest ratings of valence, but not on arousal. We address this issue in Experiment 2 by using a differently standardized stimulus sets.

## Experiment 2

Experiment 1 left several questions unanswered. One issue is whether consumption behavior can be influenced by affective pictures other than faces. This is important as faces are unique in several respects. For one, faces are extremely frequent stimuli for which people develop processing expertise (Farah et al., 1998). Further, facial expressions might influence affective responses via low-level mechanisms tuned to detection of rudimentary features (Morris et al., 1998; Vuilleumier et al., 2003). Finally, facial expressions, especially smiles and frowns, are frequently used gestures of approval or disapproval, including in the domain of consumption (Klinnert et al., 1983). To address the issue of possible specificity for facial expressions, in Experiment 2 we selected pictures from the standardized IAPS set that included emotional objects or scenes that should not receive privileged processing (e.g., dog, gun, astronaut, and dentist). We then selected words from the standardized ANEW that matched the object in the pictures and were similar in valence and arousal.

To further address the possible role of the amount of experience with the stimulus, we also manipulated frequency of words and matched pictures. Some studies suggest that priming effects are more easily obtained with high frequency words, due to their greater initial activation (McClelland and Rumelhart, 1981). Similarly, previous studies that obtained priming effects with pictures tended to use familiar, high frequency objects (e.g., Dell'Acqua and Grainger, 1999).

In addition, because in Experiment 1 we observed no effects of presentation duration, all primes in Experiment 2 were presented supraliminally, but unobtrusively. Making both picture and word primes fully visible also reduces the possibility that any differences in their influence have to do with the relative salience and complexity of the word and picture stimuli.

Finally, one potential methodological concern about Experiment 1 is that the face classification task (male/female) was different from the word classification task (object/animal). It is possible that word classification along the object-animal dimensions is more difficult than face classification along the gender dimension. This could result in more distraction from the affective meaning of the word stimuli relative to the face stimuli, or deeper, more careful processing of word stimuli. To address this concern, in Experiment 2 participants judged all stimuli on the same dimension—whether they contained a living or a non-living object.

### Method

#### Participants and procedure

Forty-eight undergraduates (12 males, 36 females, mean age = 20.7 years) participated for extra credit. The cover story, the equipment, the order of events, and the general procedure were similar to Experiment 1, except for the classification task. As shown in Figure 1 (bottom panel), the affective prime was shown for 200 ms, which participants classified on the living/non-living dimension. Because the primes were always supraliminal, we eliminated the pre-mask cross as well as the neutral post-mask. Eight priming trials were arranged in four within-subjects counterbalanced blocks that were: (i) positive and high frequency, (ii) positive and low frequency, (iii) negative and high frequency, and (iv) negative and low frequency. Format of prime stimulus (words or pictures) was manipulated between subjects, with half the subjects in the words condition and half in the picture condition.

##### Word stimuli

Word stimuli as well as their valence, arousal, and frequency ratings came from the ANEW set by Bradley and Lang (1999) and are listed in the Appendix 1 (right panel). Half of the words represented living things and half represented non-living things. Words ranged in width from 8 to 15 cm and were all 2.5 cm high.

##### Picture stimuli

Picture stimuli along with their valence and arousal ratings came from IAPS set by Lang et al. (2005) and are listed in the Appendix 1 (right panel). Half of the pictures represented living things and half represented non-living things. Picture stimuli were presented in a full screen view on a 21-inch (48-cm) computer monitor.

### Results

We analyzed how prime valence (positive vs. negative), prime format (faces vs. words), and prime frequency (low vs. high) influenced consumption behavior, subjective experience, and drink ratings. Data underlying these analyses are available under the following link: http://pages.ucsd.edu/~pwinkiel/koolaid-picture-words.zip. Means are presented in Table 2.

**Table 2:** Means and standard errors of critical dependent measures in Experiment 2.

**VALENCE**	**Positive**				**Negative**			
**FORMAT**	**Pictures**		**Words**		**Pictures**		**Words**	
**FREQUENCY**	**High**	**Low**	**High**	**Low**	**High**	**Low**	**High**	**Low**
**BEHAVIOR**								
Pouring	39.71	54.17	35.54	40.96	37.33	42.96	48.29	45.00
	*5.70*	*8.13*	*5.70*	*8.13*	*7.18*	*5.69*	*7.18*	*5.69*
Drinking	24.88	43.92	25.00	28.25	24.63	26.75	34.04	28.54
	*4.93*	*8.18*	*4.93*	*8.18*	*6.60*	*4.75*	*6.60*	*4.75*
**EXPERIENCE**								
Mood	1.29	0.83	0.88	0.88	0.88	0.83	0.83	0.88
	*0.42*	*0.40*	*0.42*	*0.40*	*0.43*	*0.40*	*0.43*	*0.40*
Arousal	0.08	−0.13	−1.17	−1.21	0.21	0.13	−0.92	−1.29
	*0.48*	*0.49*	*0.48*	*0.49*	*0.50*	*0.49*	*0.50*	*0.49*
**DRINK RATINGS**								
Delicious	5.63	5.25	6.08	5.21	5.29	6.04	5.38	5.08
	*0.43*	*0.51*	*0.43*	*0.51*	*0.47*	*0.45*	*0.47*	*0.45*
Wanting	1.29	1.25	1.38	1.17	1.13	1.50	1.21	1.00
	*0.20*	*0.19*	*0.20*	*0.19*	*0.18*	*0.18*	*0.18*	*0.18*
Paying	0.44	0.45	0.47	0.40	0.42	0.48	0.42	0.40
	*0.05*	*0.05*	*0.05*	*0.05*	*0.05*	*0.05*	*0.05*	*0.05*
Sweet	5.75	5.50	5.79	5.63	5.54	5.92	5.71	5.96
	*0.44*	*0.45*	*0.44*	*0.45*	*0.44*	*0.46*	*0.44*	*0.46*

*Consumption behavior was measured in grams. Experience on −5 to + 5 scale (not at all, very much). Ratings of deliciousness and sweetness on 0–10 scale. Wanting on 0–6 scale reflecting desired amount, and paying on 10 cents to 1 dollar scale*.

#### Consumption behavior: pouring and drinking

Priming effects on consumption behaviors (pouring and drinking) were analyzed with a four-way mixed MANOVA with three within-subject factors—type of behavior (pouring vs. drinking), prime valence (positive vs. negative), and prime frequency (high vs. low), as well as the between-subjects factor of prime format (pictures vs. words). There was a theoretically uninteresting main effect for behavior type, showing that participants poured more than they drank, *F*_(1, 46)_ = 31.26, *p* < 0.001. More interestingly, there was a prime format by valence interaction, *F*_(1, 46)_ = 6.87, *p* < 0.05. This reflects that positive vs. negative stimuli had different impact on consumption behaviors as a function of the type of prime.

Though we did not observe an interaction with the behavior type in this analysis (but see below), we wanted to further probe the nature of these effects on the individual measures of pouring and drinking. The results are illustrated in Figure 2 (right panel). On pouring, there was a valence by stimulus format interaction, *F*_(1, 46)_ = 5.82, *p* < 0.05. Participants tended to pour more after priming with positive pictures than with negative pictures (*p* = 0.13), but *less* after priming with positive words than with negative words (*p* < 0.07). There was also a valence by stimulus format interaction on drinking, *F*_(1, 46)_ = 6.65, *p* < 0.05. Participants drank more after priming with positive pictures than with negative pictures (*p* < 0.05). They also tended to drink *less* after priming with positive words than with negative words, but this effect was far from significance (*p* = 0.21).

Finally, like in Experiment 1, we examined possible thirst effects by dividing participants into high-thirst and low-thirst groups (via median split) and adding this factor to the analysis. On pouring and on drinking, the effects of valence did not vary by the level of thirst. For completeness, we also conducted a full, five-way MANOVA with behavior type, prime valence, frequency, prime format, and thirst level. This analysis revealed a complex 4-way interaction, *F*_(1, 44)_ = 4.12, *p* = 0.048. However, diagnosing this interaction revealed that it was primarily driven by different impact of stimulus frequency across different levels of other factors, so we will not decompose it here as it is not theoretically relevant. Likewise, the analysis with thirst as a continuous measure yielded no significant correlation between participants' thirst and the impact of primes on pouring or drinking.

#### Ratings of subjective experience and ratings of drinks

For ratings of subjective experience and ratings of drinks, no significant effects were revealed by a three-way MANOVA of prime valence, prime stimulus format, and frequency. There was a marginal 2-way interaction between valence and stimulus format on deliciousness, *F*_(1, 46)_ = 3.98, *p* < 0.06, but no simple effects were significant and this interaction was not obtained on other ratings.

### Discussion

The main results of Experiment 2 were similar to Experiment 1. Priming with emotional pictures influenced consumption behavior in a valence-congruent fashion, with increased consumption after priming with positive than negative pictures. In comparison, priming with valence-matched words yielded trends in the opposite, valence-incongruent direction, with *decreased* consumption after positive than negative words. Similar to Experiment 1, priming had no significant effect on either subjective experience (mood and arousal) or on drink ratings. Interestingly, the frequency of the stimuli did not significantly modify the effects. Finally, unlike Experiment 1, there were no significant effects of thirst, even on priming with emotional pictures.

## General discussion

The main finding of the current experiments is that unobtrusive exposure to emotional facial expressions (Experiment 1) and emotional pictures (Experiment 2) influenced participants' consumption behavior in a valence-congruent way. In comparison, the exposure to emotional words tended to lead to the opposite, valence-incongruent contrast effects. However, only one of those contrast effects was significant and required that participants were thirsty (Experiment 1). The divergent patterns of influence were obtained even though words were matched on valence with pictures of emotional facial expressions (Experiment 1), and on valence and arousal with pictures of emotional objects (Experiment 2). Before we discuss the implications of this main finding, let us summarize and discuss some secondary findings.

Several potentially interesting effects were statistically non-significant. Without treating these non-significant results as evidential, we point out their potential relevance. In Experiment 1 the effect of presentation duration (subliminal vs. supraliminal, but unobtrusive) was non-significant. The non-significant effect of duration is consistent with a range of effects in this literature (including quite small ones) suggesting that priming direction is determined not by awareness of the prime *per se*, but by awareness of the prime influence (Bargh, 1992). It also matches some neuroscience data which found little difference between subliminal and supraliminal, but unobtrusive presentations of facial expressions on limbic activation (Whalen et al., 1998; Critchley et al., 2000). The non-significant effect of presentation duration reduces the concern that any differences between pictures and words were due to sheer perceptibility. In Experiment 2, the stimulus frequency effect was also statistically non-significant. This non-significant effect fits the notion that the relative amount of participants' experience with words and pictures stimuli is not the reason for their different influence.

In both experiments, we found no significant effects of stimulus valence on subjective experience, even for participants who showed reliable valence effects on the consumption behavior. This finding fits earlier reports that affective stimuli, can influence behavior without eliciting changes in conscious experience (Winkielman et al., 1997, 2005; Zemack-Rugar et al., 2007; Bornemann et al., 2012). Theoretically, this result fits the idea of “unconscious emotion” (Winkielman et al., 2011; Smith and Lane, 2016). However, this claim requires much further testing because phenomenal changes could be revealed with more sensitive measures of affective experience (Schooler et al., 2015), under conditions that promote the emergence of state awareness (Morsella, 2005), or in participants attuned to their bodily state (Bornemann et al., 2012). Clearly, it is also possible that accumulating data will reveal support for this null hypothesis (i.e., Bayes factor favoring the null). At this point, any conclusion about “unconscious emotion” in this paradigm is tentative.

Finally, consistent with earlier research, participants' motivational state—thirst—modified the impact of facial expressions on consumption (Winkielman et al., 2005). However, we found no statistically significant effect of thirst on the impact of emotional IAPS pictures containing objects and scenes. This non-significant finding was unexpected and could simply reflect differences in baseline thirst levels or drinking levels across studies, or overall levels of task motivation (Gendolla, 2000). Unfortunately, in the current research we did not compare facial expressions and IAPS in the same experiment. But, if such findings were confirmed by future studies, they could be interpreted in light of arguments that facial expressions have an advantage in modulating low-level affective and motivational mechanisms (Whalen et al., 1998; Vuilleumier et al., 2003). Consistently, faces are good triggers of “approach-avoidance” tendencies (Klinnert et al., 1983; Marsh et al., 2005; but see Phaf et al., 2014). Future research may explore this possibility.

### Pictures and words

Our main finding was the valence-congruent influence of affective pictures, but not words, on spontaneous behavior. Two theoretical accounts may provide explanation for this finding.

One interpretation is offered by the framework of grounded or embodied cognition (Barsalou, 1999). As discussed in the introduction, this framework assumes that processing of affective stimuli can be purely conceptual, or can be accompanied by somatosensory reactions (Niedenthal, 2007; Winkielman et al., 2015b). If an incidental stimulus elicits a somatosensory reaction, and the mechanisms underlying a particular behavior draw on the same set of somatosensory resources, one should observe a congruent influence (Winkielman et al., 2007; Knutson et al., 2008). This interpretation is consistent with existing literature, discussed earlier, showing that viewing pictures of emotional faces or emotional scenes leads to greater physiological effects than viewing emotion words (Harris et al., 2003; Larsen et al., 2003).

Why do emotional pictures exert this influence but not words? One possibility is that the evolutionarily “old” affect system is particularly suited for dealing with pictorial information. A picture of a smiley face, a juicy cake, a slithering snake, or a bloody knife may be quickly interpreted by early visual areas connected to subcortical structures involved in generation of physiological responses (de Gelder et al., 2011). In comparison, words are polysemous—even simple nouns like “cake” or “gun” can appear in contexts that flip its affective meaning (cake=dung, guns=biceps). So, before a word is able to elicit a robust physiological response, it needs to be processed more deeply by more advanced mechanisms, and perhaps even “translated” into a specific sensory representation (Paivio, 1971; Vandenberghe et al., 1996; Naccache et al., 2005; Niedenthal et al., 2009). Furthermore, without the physiological response to guide subsequent processing in a valence-congruent fashion, words might be subject to more “cold” cognitive operations.

A different theoretical perspective suggests that the power of affective pictures comes not from their ability to trigger physiological responses, but from their privileged access to the cognitive semantic system (Potter et al., 1986; Glaser, 1992), including the semantic network representing evaluative information (De Houwer and Hermans, 1994). Consistent with this assumption, some studies found stronger impact of pictures and words in a fast evaluative priming task (Spruyt et al., 2002, but see Kiefer et al., 2017). However, this “privileged semantic access”, unlike the “embodiment” explanation, does not predict greater impact of pictures on consumption behavior—a finding we observed. It also predicts greater impact of primes on evaluation of drink qualities and evaluation of one's mood—findings we failed to observe. Further, the privileged semantic access account would predict greater impact of supraliminal, than subliminal words, and high-frequency than low-frequency words—again, findings that we failed to observe. On the other hand, this verdict against this explanation needs to be tentative until future studies directly examine the relative availability of semantic concepts after picture and word priming and the relative sensitivity of behavioral and self-report measures.

More generally, the privileged semantic access explanation, may on the first glance seem contradictory to the embodied explanation. However, it can be seen as compatible. After all, theories of embodied cognition propose that semantic processing is supported by somatosensory simulation involving “perception-like” processes. In fact, categorization literature suggests that when participants try to access a deep meaning of an emotional concept, they generate concrete images and develop a specific “situated” conceptualization (Barsalou, 1999; Niedenthal et al., 2005; Oosterwijk et al., 2015). However, that simulation process takes motivation, effort, and time (Wilson-Mendenhall et al., 2011). From that perspective, pictures offer “pre-packaged” situated conceptualizations—an idea which explains their privileged access and greater spontaneous impact on physiology.

One weakness of both the embodiment and the “privileged access” explanations is that they both predict that the impact of words should simply be weaker primes than pictures. However, even though most of the individual effects were not significant, a general tendency was for words to have an opposite effect. One speculative explanation is that because our words were concrete nouns, referring to specific exemplars rather than general traits, they might have triggered comparison contrast operations, thus creating a response tendency opposite to the prime implications (Dijksterhuis et al., 1998). On the other hand, emotional pictures also represent concrete exemplars, albeit their exemplar status might be qualified by a broad affective activation.

Finally, it is important to acknowledge that words can be powerful triggers of affect and actions. Occasionally, a word may be worth a thousand pictures. Humans are a symbolic species and much of their affairs, including those of the heart, take root and express in symbolically-mediated interactions (e.g., via political manifestos, love letters, or arguments presented in journal articles). Words can do that because they “invite and guide” us to develop mental models that real images may not match (Barsalou, 1999; Bergen, 2012). As a result, words can trigger powerful emotional reactions, with all their physiological consequences. Some of them require a deep understanding of the implications of what has been said (“when you left house today, you forgot to turn off the hot iron”). Other words, such as taboo words, terms of endearment, or native-language emotional terms can work directly through previously pre-computed meanings (Harris et al., 2003; Baumeister et al., 2017). Future research may focus on specifying different conditions under which words and pictures are influential as affective primes of judgment and action, and explore more precisely the underlying psychological and physiological mechanisms. In any case, we hope that the words and pictures presented in this article offer an initial contribution toward this goal, and help us move toward better integration of theories of cognition and emotion.

## Ethics statement

This study was carried out in accordance with the recommendations of UCSD Human Research Protection Program with written informed consent from all subjects. All subjects gave written informed consent in accordance with the Declaration of Helsinki. The protocol was approved by the HRPP human subject committee.

## Author contributions

YG collected, entered, did preliminary analyses of the data and described the procedures and methods. PW conceptualized the study and wrote the introduction, results, and discussion sections.

### Conflict of interest statement

The authors declare that the research was conducted in the absence of any commercial or financial relationships that could be construed as a potential conflict of interest.

## Footnotes

^1^To avoid strong claims about the unconscious, Murphy and Zajonc (1993) used the terms “suboptimal” to refer to very brief presentation conditions. In this paper, we treat the terms “subliminal” and “suboptimal” as equivalent, but also wish to avoid making strong claims about the existing literature and our own presentation conditions.

^2^Preliminary analyses revealed no effects of the blocks order, so main analyses collapsed across this variable.

## Appendix

**Table:**

**EXPERIMENT 1**				**EXPERIMENT 2**								
**WORDS**		**FACES**			**WORD(anew)**				**PICTURES (iaps)**			
**Name**	**Val**.	**Code**	**Val**.			**Code**	**Val**.	**Aro**.	**Freq**	**Code**	**Val**.	**Arou**.
**POSITIVE**				**POS**								
Dog	6.64	haf1	6.31	FREQ	Baby	31	8.22	5.53	62	2058	7.91	5.09
Kitten	6.52	haf2	6.56		Bride	670	7.34	5.55	33	2209	7.64	5.59
Panda	6.52	ham1	5.98		Candy	61	6.54	4.58	16	7410	6.91	4.55
Pony	6.06	ham3	6.57		Cash	503	8.37	7.37	36	8502	7.51	5.78
Doll	6.63	hcf1	6.5		Dog	511	7.57	5.76	75	1500	7.24	4.12
Pie	6.41	hcf2	6.38		Fish	559	6.04	4	35	1900	6.65	3.46
Wine	5.91	hcm1	6.76		Gold	191	7.54	5.76	52	8500	6.96	5.6
Fragrance	6.07	hcm2	6.32		Wine	496	5.95	4.78	72	7280	7.2	4.46
Mean	6.35	mean	6.42		Mean		7.20	5.42	47.63		7.25	4.83
NEUTRAL				POS	Astronaut	501	6.66	5.28	2	5470	7.35	6.02
Horse	5.71	naf1	4.92	RARE	Butterfly	58	7.17	3.47	2	1603	6.9	3.37
Owl	5.49	naf2	4.66		Diver	510	6.45	5.04	1	8280	6.38	5.05
Goat	5.06	naf3	4.83		Fireworks	513	7.55	6.67	5	5480	7.53	5.48
Sheep	5.1	naf4	5		Pizza	526	6.65	5.24	3	7352	6.2	4.58
Metal	4.85	nam1	4.81		Puppy	336	7.56	5.85	2	1710	8.34	5.41
Hammer	4.78	nam2	5.06		Sailboat	529	7.25	4.88	1	8170	7.63	6.12
Cabinet	5.05	nam3	5.01		Skyscraper	573	5.88	5.71	2	7510	6.05	4.52
Column	5.17	nam4	4.89		Mean		6.90	5.27	2.25		7.05	5.07
Cow	5.37	ncf1	5.04	NEG	Bees	583	3.2	6.51	15	1390	4.5	5.29
Donkey	5.31	ncf2	4.91	FREQ	Bomb	46	2.1	7.15	36	9630	2.96	6.06
Goose	5.47	ncf3	5		Cemetery	65	2.63	4.82	15	9000	2.55	4.06
Lamb	5.36	ncf4	4.91		Criminal	705	2.93	4.79	24	6243	2.33	5.99
Taxi	5	ncm1	5.13		Dentist	589	4.02	5.73	12	9584	3.34	4.96
Ink	5.03	ncm2	4.88		Gun	593	3.47	7.02	118	6610	3.6	5.06
Iron	4.72	ncm3	5		Knife	596	3.62	5.8	76	9401	4.53	3.88
Hairdryer	4.82	ncm4	4.81		Snake	609	3.31	6.82	44	1050	3.46	6.87
Mean	5.14	mean	4.93		Mean		3.16	6.08	42.50		3.41	5.27
**NEGATIVE**				**NEG**								
Leech	3.38	aaf1	3	RARE	Addict	581	2.48	5.66	1	2710	2.52	5.46
Rat	3.44	aaf2	3.31		Alcoholic	582	2.84	5.69	3	2753	3.17	4.29
Worm	3.38	aam1	3.19		Garbage	182	2.98	5.04	7	9340	2.41	5.16
Snake	3.88	aam2	4		Narcotic	894	4.29	4.93	2	9101	3.62	4.02
Knife	3.62	acf1	3.44		Roach	363	2.35	6.64	2	7380	2.46	5.88
Needle	3.82	acf2	3.75		Spider	610	3.33	5.71	2	1220	3.47	5.57
Tobacco	3.28	acm1	3.49		Tornado	444	2.55	6.83	1	5971	3.49	6.65
Crutch	3.4	acm2	3.82		Vomit	481	2.06	5.75	3	9320	2.65	4.93
mean	3.53	mean	3.5		mean		2.86	5.78	2.63		2.97	5.25
